# Psychometric validation and meaningful change thresholds of the Worst Itching Intensity Numerical Rating Scale for assessing itch in patients with chronic kidney disease-associated pruritus

**DOI:** 10.1186/s41687-021-00404-z

**Published:** 2021-12-24

**Authors:** Margaret K. Vernon, Laura L. Swett, Rebecca M. Speck, Catherine Munera, Robert H. Spencer, Warren Wen, Frédérique Menzaghi

**Affiliations:** 1 Evidera, Bethesda, MD USA; 2 Cara Therapeutics, Inc., 4 Stamford Plaza, 107 Elm Street, 9th Fl., Stamford, CT 06902 USA

**Keywords:** Chronic kidney disease, Numeric rating scale, Patient-reported outcome measures, Pruritus, Psychometrics

## Abstract

**Background:**

Chronic kidney disease-associated pruritus (CKD-aP) is characterized by persistent itch that often leads to substantially impaired quality of life. The Worst Itching Intensity Numerical Rating Scale (WI-NRS) is a single-item patient-reported outcome measure in which patients indicate the intensity of the worst itching they experienced over the past 24 h. Here, we evaluated the content validity and psychometric properties of the WI-NRS and confirmed the threshold of meaningful change in hemodialysis patients with moderate-to-severe CKD-aP.

**Methods:**

Content validity interviews were conducted in 23 patients. Psychometric properties of the WI-NRS were assessed using data from one phase 2 (N = 174) and two phase 3 (N = 848) clinical trials investigating an anti-pruritic treatment. Anchor-based methods were used to confirm meaningful within-patient change score thresholds in the phase 3 trial patients and mixed-method exit interviews (N = 70) contributed further insight.

**Results:**

Content validity interviews indicated patients considered the WI-NRS to be straightforward, comprehensive, and relevant. Test–retest reliability was strong in both trial cohorts (intraclass correlation coefficients > 0.75). Construct validity analyses indicated high correlation between the WI-NRS and other measures of itch. Anchor-based analyses showed a reduction of ≥ 3 points from baseline score represented an appropriate clinically meaningful within-patient change on the WI-NRS. In the exit interviews, all patients with a reduction ≥ 3 points considered the change meaningful.

**Conclusions:**

The WI-NRS is a reliable, valid, and responsive measure of itch intensity for patients with moderate-to-severe CKD-aP. These results support its use to assess treatment efficacy and in clinical evaluation and management of pruritus in hemodialysis patients.

**Supplementary Information:**

The online version contains supplementary material available at 10.1186/s41687-021-00404-z.

## Plain English summary

Itching is a distressing medical condition common in patients with chronic kidney disease, especially those undergoing hemodialysis. The itch often leads to skin damage due to a continuous and uncontrollable urge to scratch. It affects about 60% of hemodialysis patients and can be severe enough to seriously affect quality of life. At present, there are no approved therapies. To evaluate whether new treatments for itch are effective, clinicians need to assess if the intensity of itch decreases over time. However, because itch intensity can only be measured accurately by the person experiencing it, a measure is required that can be easily understood and used by patients. This study evaluated a scale in which patients mark a number between ‘0’ (corresponding to no itch) and ‘10’ (the worst itching imaginable), to describe the worst itch intensity they experienced over the last 24 hours. Using data from three clinical trials of a novel treatment for itch in patients undergoing hemodialysis with moderate-to-severe pruritus, we found that the scale was reliable in repeat-testing experiments, and mirrored other methods of measuring changes in itch. In interviews, patients said they found the scale straightforward and easy to complete. Our analysis and patients’ opinions showed a 3-point reduction in itch intensity on the scale represented a meaningful improvement. These findings support the use of this scale to assess the efficacy of new treatments and in clinical evaluation and management of pruritus in patients with chronic kidney disease.

**Supplementary Information:**

The online version contains supplementary material available at 10.1186/s41687-021-00404-z.

## Background

Pruritus is one of the most common and distressing symptoms in patients with chronic kidney disease receiving hemodialysis [1–4]. Chronic kidney disease-associated pruritus (CKD-aP) does not originate from skin lesions, but rather is a systemic, persistent itch sensation that often leads to considerable mechanical skin damage due to a continuous and uncontrollable urge to scratch [5, 6]. More than 60% of patients undergoing hemodialysis have some degree of pruritus, with 20–40% suffering from moderate-to-severe pruritus [1, 7–9]. Patients with CKD-aP suffer severely impaired health-related quality of life (HRQoL), including sleep disturbance, chronic fatigue, agitation, shame, social isolation, and depression [1, 3, 7, 8, 10, 11]. Severe itching is also associated with an increased risk of mortality [7]. Despite its high prevalence and distressing sequelae, CKD-aP remains poorly characterized and has no approved treatment [8]. The pruritus tends not to be adequately controlled by topical emollients, antihistamines, or steroids or off-label used treatment, like gabapentin, which are not always well tolerated [2, 8].

Since pruritus is a symptom that only patients themselves can report on, a patient-reported outcome (PRO) measure is required to evaluate the efficacy of any new investigational treatment. Numerical Rating Scales (NRS) measuring worst itch intensity are commonly used in clinical trials, but few have had their psychometric properties evaluated in line with best practices and FDA evidentiary standards [12]. Furthermore, the magnitude of the reduction in NRS scores that represents meaningful improvement for patients with CKD-aP has not been extensively studied or established.

The Worst Itching Intensity NRS (WI-NRS) is a simple-to-use, single-item PRO [13, 14]. Patients indicate the intensity of the worst itching they have experienced over the past 24 h by marking one of 11 numbers—from 0 to 10—that best describe the worst itching experiences (“0” labeled with the anchor phrase “no itching” and “10” labelled “worst itching imaginable”). This WI-NRS has been validated for dermatologic conditions like psoriasis [15, 16] and atopic dermatitis [14] but not for systemic pruritus like CKD-aP. We previously identified that a reduction of ≥ 3 points on the WI-NRS represented a clinically meaningful response to treatment with the selective kappa opioid receptor agonist difelikefalin in hemodialysis patients with moderate-to-severe pruritus [13]. However, gaps remain in our understanding of the measure’s content validity from patients’ perspectives as well as its other psychometric properties, including test–retest reliability and whether it mirrors other methods of measuring changes in itch (i.e., known-groups validity).

The FDA’s Patient-Focused Drug Development Guidance suggests the use of mixed methods (quantitative and qualitative) to triangulate on defining meaningful within-patient change thresholds for clinical outcome assessments (COA) [17]. While there is guidance on quantitative approaches to determine meaningful within-patient change thresholds (anchor-based methods are preferred) [18, 19], there is no consensus on optimal methods for qualitative or other mixed-methods approaches. An emerging approach for evaluating meaningful within-patient change thresholds for COAs is to survey or interview patients as they exit a clinical trial to ascertain their experience of treatment, whether the change they experienced was meaningful, and to gather further interpretation of score changes on administered COA endpoints [20–22].

Thus, the goal of the present study was to evaluate the content validity and psychometric properties of the WI-NRS in hemodialysis patients with CKD-aP based on qualitative interviewing and quantitative methodologies, as well as to confirm our earlier estimated meaningful change threshold [13] using anchor-based analyses and mixed methods exit interviews.

## Methods

### Content validity methods

Content validity of the WI-NRS (see Additional file 1: Fig. S1) was evaluated through qualitative interviews with hemodialysis patients with CKD-aP of any severity. Interview participants were recruited from four dialysis centers in the US, had to be aged ≥ 18 years, on hemodialysis three times per week for ≥ 3 months before screening, self-reporting pruritus ≤ 1 month before screening, and could not have pruritus unrelated to CKD, pruritus only during dialysis sessions, or a co-morbidity that might compromise the patient, study, or study measures. The content validity interviews included concept elicitation questions to ensure participants’ descriptions of their CKD-aP were consistent with the WI-NRS content and wording, and standardized cognitive interviewing to ensure that the wording, response options, and recall period were appropriate for capturing patients’ experiences. Interviews were conducted in English following a semi-structured interview guide, took approximately 60 min, and were digitally audio-recorded with the consent of the participants. Transcripts were analyzed using ATLAS.ti (version 7.5.12 or higher). After the first five interviews, a high-level qualitative analysis determined that no modifications to the WI-NRS was required.

### Psychometric analyses

Psychometric properties of the WI-NRS were assessed using data collected from one phase 2 [23], and two phase 3 (US-based KALM-1 and global KALM-2) [24, 25] randomized placebo-controlled multicenter studies investigating the safety and efficacy of intravenous difelikefalin in patients with moderate-to-severe pruritus undergoing hemodialysis. The phase 2 dataset was used to assess psychometric validity. Pooled phase 3 trial data were used for confirmatory analyses and in an anchor-based analysis to verify the meaningful change threshold previously established with phase 2 data [13]. Eligibility criteria for patients in the phase 2 (N = 174) and phase 3 (N = 848) trials were similar to the content validity interviews, although patients were additionally required to self-report baseline pruritus severity of ≥ 4 on the WI-NRS (calculated as the average of the daily WI-NRS scores collected over a 7-day run-in period) [23–25]. WI-NRS data were analyzed as weekly mean scores, defined as the average of the daily ratings for each week from baseline to the last week of the treatment period. For a weekly score to be calculated, data had to be available for ≥ 4 of 7 days, otherwise the weekly score was set to missing. Table 1 details other PRO measures from the phase 2 and phase 3 studies used in the psychometric analyses. Psychometric assessments were evaluated in line with the US Food and Drug Administration guidance on PROs [12]. Statistical analyses were conducted using SAS version 9.4 and used a 2-sided significance level of *P* < 0.05.

**Table 1:** Patient-reported outcome measures

Measure	Response scale	Recall period	References
*WI-NRS* (Worst Itching Intensity Numerical Rating Scale)	Worst itching experienced on 11-point scale: 0–10. “0” labelled with the anchor phrase “no itching” and “10” labelled “worst itching imaginable”	24 h	[13, 14]
*Skindex-10*	10 questions with a 7-point scale: 0–6. “0” labelled with the anchor phrase “never bothered” and “6” labelled “always bothered”. Total score is sum of the numeric value of each answered question (range, 0–60). Total score is subdivided into three domain scores that are sums of the scores of the following questions: disease domain (questions 1–3 with score range from of 0 to 18); mood/emotional distress domain (questions 4–6 with score range of 0–18); and social functioning domain (questions 7–10 with score range of 0–24)	Past week	[8]
*5-D itch*	Five dimensions assessed: degree (k = 1), duration (k = 1), direction (k = 1), disability (k = 4), and distribution (k = 16). Degree, duration, direction and disability domains measured by a five-point Likert scale with higher scores reflecting worse itch. Disability domain includes four items that assess itching impact on daily activities: sleep, leisure/social activities, housework/errands and work/school; disability domain score is highest score on any of the four items. For distribution domain, number of affected body parts is tallied (sum, 0–16) and the sum sorted into five scoring bins: sum of 0–2 = score of 1, sum of 3–5 = score of 2, sum of 6–10 = score of 3, sum of 11–13 = score of 4, and sum of 14–16 = score of 5. Scores of each of the five domains summed together to obtain a total 5-D score ranging from 5 (no pruritus) to 25 (most severe pruritus)	Past 2 weeks	[26]
*Patient self-categorization of pruritus severity*	Patients asked to select which of three patient profiles they are most like according to occurrence of scratch marks on skin, problems sleeping because of itching, and feelings of agitations or sadness: Patient A (mild signs and symptoms), Patient B (moderate signs and symptoms), or Patient C (severe signs and symptoms)	Current	[8]
*MOS sleep scale*	For most questions, 6-point scale: 1–6. “1” labelled with the anchor phrase “all of the time” and “6” labelled “none of the time” indicating the frequency of various aspects of sleep disruption. Instructions also provided to estimate average hours of sleep during the past week and length of time taken to fall asleep. Sleep Problem Index II (k-9; items 1, 3, 4, 5, 6, 7, 8, 9, 12), Sleep Problem Index I (k-6; items 4, 5, 7, 8, 9, 12), and Sleep Disturbance (k = 4; items 1, 3, 7, 8) subscales can also be calculated. Higher scores reflect better sleep-related HRQoL	Past week	[27]
*PGI-S* (Patient Global Impression of Worst Itch Severity)	Assesses patient impression of itch severity. Single-item scale with five possible values ranging from none to very severe; higher scores reflect worse severity	24 h	[28]
*PGI-C* (Patient Global Impression of Change)	Assesses patient impression of change (improvement or worsening) in overall status relative to the start of the study. Single-item measure with values ranging from ‘1’ (Very Much Improved) to ‘7’ (Very Much Worse); higher scores reflect worse status	Current vs. earlier time point	[28]
*M-PGIC* (modified Patient Global Impression of Change)	Assesses patients’ overall impression of change in itch during the course of the clinical trial and whether the amount of improvement was meaningful to them. Brief, one-item measure with four response options: “My itch got worse,” “No change,” “My itch got better but the amount of improvement was not meaningful to me,” and “My itch got better and the amount of improvement was meaningful to me”	Current vs. earlier time point	[28]

Missing data were handled according to the instructions provided by the instrument authors. Abbreviations: HRQoL, health-related quality of life; MOS, Medical Outcomes Study

#### Test–retest reliability

For the phase 2 cohort, test–retest reliability was assessed by determining intraclass correlation coefficients (ICCs) between Weeks 1 and 2 and between Weeks 2 and 4, based on the ICC(2,1) method [29]. Patients with the same Patient Global Impression of Worst Itch Severity (PGI-S) response between the test and retest time points were defined as stable and included in the analysis. For the phase 3 cohort, test–retest reliability was assessed using the same time points with all evaluable patients included. As generally accepted [30, 31], test–retest reliability was supported with ICCs > 0.70.

#### Construct validity

The construct validity of the WI-NRS was assessed by examining convergent and divergent validity. Moderate (r ≥ 0.3 to < 0.5) or large (r ≥ 0.5) convergent correlations by Cohen’s standards [32] were hypothesized for the PGI-S (phase 2 only) and for items within the Skindex-10 and the 5-D Itch that measure similar concepts to the WI-NRS. The MOS Sleep Scale domain scores were used for divergent validity tests on the phase 2 data (i.e., to assess the extent to which sleep and itch, which are less related concepts, exhibit low correlations [r < 0.3] with one another).

#### Known-groups validity

To assess the discriminant properties of the WI-NRS, known groups validity was evaluated by creating groups using the PROs collected from the phase 2 study (PGI-S, Patient Self-categorization of Pruritus Disease Severity, Skindex-10, 5-D Itch, MOS Sleep Problem Index II) and the pooled phase 3 studies (Skindex-10, 5-D Itch). The mean of the screening (i.e., baseline) WI-NRS was computed for each category of each PRO measure. As the data were normally distributed (by Kolmogorov–Smirnov test), a linear model analysis of variance (ANOVA) was conducted with the baseline weekly mean WI-NRS as the dependent variable and the categorical known group as the independent variable (separate models for each individual known group) to evaluate differences in weekly mean WI-NRS scores. Two-sample t-tests were used to compare differences in WI-NRS for known groups with two categories; linear model ANOVA were used for known groups with more than two categories.

### Meaningful change threshold study and analysis

The anchor-based methods and meaningful change threshold for the phase 2 cohort have been previously published [13]. The same anchor-based approach was used to define the point-change on the WI-NRS (change from baseline to end of treatment) that represented a clinically meaningful improvement to patients in the pooled phase 3 cohort. The Patient Global Impression of Change (PGI-C) was used as the anchor; this FDA-recommended [33] measure specifically asks patients to indicate the improvement of their condition taking into consideration treatment effect and patient expectation. The “minimally improved,” PGI-C anchor category was used in the primary anchor approach. The “minimally improved” and “much improved” categories were combined for use as a secondary anchor.

### Exit study to further evaluate threshold of meaningful change

To determine what constituted a meaningful change from patients’ perspectives, mixed-method exit interviews were conducted with patients completing the phase 3 trials using methodologies adapted from Koochaki et al. [21] and McCarrier et al. [20]. For the exit interviews, eligible patients had to complete the final visit of the 12-week double-blind treatment period of either phase 3 trial. Enrollment to the exit interviews was stratified to ensure different point change ranges on the WI-NRS were represented: 10–12 patients reporting a one-point improvement and 15–20 reporting a two-, three-, and four-point improvement on the WI-NRS from baseline to Week 8–10. Exit interviews involved one-on-one, telephone-based interviews in either English or Spanish. Interviews lasted 60–90 min, and were conducted using a semi-structured interview guide. Participants were asked to complete the modified Patient Global Impression of Change (M-PGIC) measure (see Table 1) to evaluate whether the change in itch they experienced during the trial was meaningful to them, with a qualitative discussion of why they considered the change meaningful. Patients were then asked to review the WI-NRS and their WI-NRS change score recorded in the clinical trial (end-of-study weekly mean – baseline weekly mean), with discussion of whether that change was or was not meaningful. Distribution of WI-NRS change scores and % changes were analyzed by M-PGIC category and by participant responses on meaningful change.

## Results

### Content validity

Twenty-three interviews assessing content validity were conducted between June and August 2016 across four US sites: New York (n = 4, 17.4%), Florida (n = 5, 21.7%), California (n = 8, 34.8%), and Tennessee (n = 6, 26.1%). Participants had a mean age of 55.4 ± 17.0 years and most were White (n = 10, 43.5%), male (n = 14, 60.9%), and not Hispanic (n = 15, 65.2%) (Table 2). During concept elicitation, "itch" or "itching" were the terms most commonly used to describe CKD-aP. When asked about itch intensity and severity, many participants (n = 12, 52.2%) spontaneously provided a numerical response on a 0–10 severity scale. Some (n = 6, 26.1%) rated their itching as at least a “6” or “7” on a 1–10 or 0–10 scale. One participant (4.3%) rated their itching severity as “8–10” at night, but “5” during the day. Concept elicitation results were consistent with WI-NRS item wording and supportive of the response scale. Overall, the cognitive interviewing results showed that participants provided positive feedback on the WI-NRS and reported that the questionnaire was straightforward, comprehensive, and relevant to their experiences with CKD-aP. In addition, the instructions, wording, and response options were well understood by participants. They were able to easily select a response option and describe how they arrived at their answers. Based on a detailed review of the data, no changes to the WI-NRS were recommended.

**Table 2:** Patient characteristics

Characteristic	Content validation cohort (N = 23)	Psychometric evaluation		Exit interview cohort (N = 70)
		Phase 2 cohort (N = 174)	Pooled phase 3 cohort (N = 848)	
Age (years)				
Mean (SD)	55.4 (17.0)	57.3 (12.5)	58.7 (12.9)	55.7 (12.1)
Median [range]	61.0 [25.0–82.0]	59.0 [26.0–84.0]	59.0 [22.0–88.0]	57.0 [24.0–79.0]
Gender, n (%)				
Male	14 (60.9)	105 (60.3)	504 (59.4)	46 (65.7)
Female	9 (39.1)	69 (39.7)	344 (40.6)	24 (34.3)
Race, n (%)				
White	10 (43.5)	62 (35.6)	515 (60.7)	42 (60.0)
Black or African American	6 (26.1)	102 (58.6)	248 (29.2)	20 (28.6)
Asian	1 (4.3)	4 (2.3)	45 (5.3)	3 (4.3)
American Indian or Alaskan native	1 (4.3)	4 (2.3)	13 (1.5)	1 (1.4)
Native Hawaiian or other Pacific Islander	0 (0.0)	1 (0.6)	10 (1.2)	2 (2.9)
Other	8 (34.8)	0 (0.0)	14 (1.7)	2 (2.9)
Not reported	0 (0.0)	1 (0.6)	3 (0.4)	0 (0.0)
Ethnicity, n (%)				
Not Hispanic or Latino	15 (65.2)	136 (78.2)	572 (67.5)	32 (45.7)
Hispanic or Latino	8 (34.8)	36 (20.7)	268 (31.6)	38 (54.3)
Not reported	0 (0.0)	2 (1.1)	8 (0.9)	0 (0.0)
Years on hemodialysis mean (SD)	5.4 (5.1)	5.8 (4.7)	4.8 (4.3)	3.3 (2.3)
Years with CKD-aP, mean (SD)	2.9 (3.0)	4.4 (4.1)	3.3 (3.7)	2.7 (2.1)
Baseline WI-NRS, n (%)				
≥ 0 to < 4	3 (13.0)	–	–	–
≥ 4 to < 6	3 (13.0)	51 (29.3)	187 (22.1)	7 (10.0)
≥ 6 to < 8	11 (47.8)	81 (46.6)	384 (45.3)	34 (48.6)
≥ 8 to 10	6 (26.1)	42 (24.1)	277 (32.7)	29 (41.4)

SD, Standard deviation

### Psychometric validation

Demographics of the phase 2 and pooled phase 3 cohorts are given in Table 2.

#### Test–retest reliability

Patients from the phase 2 trial that were stable on the PGI-S had good reproducibility on their weekly mean WI-NRS scores between Week 1 and Week 2 (ICC = 0.76) and between Week 2 and Week 4 (ICC = 0.81) (Additional file 1: Table S1). WI-NRS scores for patients from the pooled phase 3 trials were also reproducible, with ICC = 0.80 between Week 1 and Week 2 and ICC = 0.81 between Week 3 and Week 4. The values were above the generally accepted 0.7 threshold [30] supporting the test–retest reliability of the WI-NRS.

#### Construct validity

WI-NRS scores significantly correlated with the Skindex-10 and 5-D Itch measures in both phase 2 and phase 3 datasets, especially with the conceptually related Skindex-10 Disease domain (r = 0.7–0.8) and the 5-D Itch Degree domain (r = 0.65–0.67) at the end of treatment (Table 3). Similarly, the weekly mean WI-NRS from the phase 2 trial patients was significantly correlated with the conceptually related PGI-S scale at the end of treatment (r = 0.63). Overall correlations were better at the end of treatment than at baseline, most likely due to higher score variance at this timepoint (to be randomized, subjects had to report WI-NRS ≥ 4 at screening). For the phase 2 trial patients, as hypothesized, correlations with the conceptually unrelated domains of the MOS Sleep measure (Sleep Problem Index I and II, and Sleep Disturbance) were small (r = 0.16–0.26) by Cohen’s standards [32].

**Table 3:** Construct validity

Comparator measure	Domains	Pearson correlation with WI-NRS			
		Baseline^a^		End of treatment	
		Pearson r	*P*-value	Pearson r	*P*-value
Phase 2 cohort					
5-D itch	Total score	0.31	< 0.0001	0.71	< 0.0001
	Degree	0.30	< 0.0001	0.67	< 0.0001
	Duration	0.22	< 0.01	0.46	< 0.0001
	Direction	0.12	NS	0.51	< 0.0001
	Disability	0.33	< 0.0001	0.56	< 0.0001
	Distribution	0.14	NS	0.37	< 0.0001
Skindex-10	Total score	0.32	< 0.0001	0.67	< 0.0001
	Disease domain	0.34	< 0.0001	0.80	< 0.0001
	Mood-emotional distress	0.35	< 0.0001	0.61	< 0.0001
	Social functioning	0.21	< 0.01	0.48	< 0.0001
PGI-S	–	0.29	< 0.001	0.63	< 0.0001
MOS sleep	Sleep Problem Index I	0.20	< 0.01	0.18	< 0.05
	Sleep Problem Index II	0.17	< 0.05	0.26	< 0.01
	Sleep Disturbance	0.16	< 0.05	0.23	< 0.01
Phase 3 cohort					
5-D itch	Total score	0.47	< 0.0001	0.70	< 0.0001
	Degree	0.41	< 0.0001	0.65	< 0.0001
	Duration	0.39	< 0.0001	0.52	< 0.0001
	Direction	0.23	< 0.0001	0.59	< 0.0001
	Disability	0.33	< 0.0001	0.53	< 0.0001
	Distribution	0.25	< 0.0001	0.43	< 0.0001
Skindex-10	Total score	0.41	< 0.0001	0.66	< 0.0001
	Disease domain	0.43	< 0.0001	0.70	< 0.0001
	Mood-emotional distress	0.37	< 0.0001	0.60	< 0.0001
	Social functioning	0.33	< 0.0001	0.54	< 0.0001

MOS, Medical Outcomes Study; NS, not significant; PGI-S, Patient Global Impression of Worst Itch Severity; WI-NRS, Worst Itching Intensity Numerical Rating Scale

^a^Pre-treatment on day 1, baseline

#### Known-groups validity

For both the phase 2 and phase 3 cohorts, the baseline WI-NRS scores were significantly different (*P* ≤ 0.032) between known groups of the conceptually related 5-D Itch total score and Skindex-10 measures (Table 4). Known-groups comparisons of WI-NRS against Patient Self-Categorization of Pruritus Disease Severity (‘Profile B’ versus ‘Profile C’) and PGI-S were also statistically significant and in the anticipated direction in the phase 2 cohort. Overall, higher (worse) mean baseline WI-NRS scores were observed for groups with worse categories defined by these independent variables. Differences in WI-NRS scores at baseline were not significantly different when grouped by the quartiles of the conceptually unrelated MOS Problem Index II (*P* = 0.1049; phase 2 cohort only).

**Table 4:** Known-groups validity of WI-NRS vs. other measures at baseline

Comparator measure	N	Mean WI-NRS score (SD)	T-value	F-value	*P*-value
Phase 2 cohort					
Self-categorization of pruritus disease severity	174		− 2.16	–	0.0324
Profile B (moderate)	123	5.8 (1.74)			
Profile C (severe)	51	6.5 (2.08)			
PGI-S	171		–	6.30	< 0.0001
None	1	4.00 (−)			
Mild	8	4.66 (1.34)			
Moderate	74	5.87 (1.64)			
Severe	74	5.96 (1.88)			
Very severe	14	8.04 (1.90)			
Skindex-10 total score	174		–	8.18	< 0.0001
≤ 25th percentile (best)	45	5.46 (1.81)			
> 25th to ≤ 50th percentile	44	5.34 (1.71)			
> 50th to ≤ 75th percentile	46	6.40 (1.59)			
> 75th percentile (worst)	39	6.97 (1.93)			
5-D Itch total score	174		–	5.96	0.0007
≤ 25th percentile (least itch)	44	5.16 (1.79)			
> 25th to ≤ 50th percentile	51	5.87 (1.54)			
> 50th to ≤ 75th percentile	41	6.49 (1.90)			
> 75th percentile (worst itch)	38	6.68 (1.96)			
MOS Sleep Problem Index II at week 1	174		–	2.08	0.1049
≤ 25th percentile (worst sleep)	46	5.71 (2.04)			
> 25th to ≤ 50th percentile	42	5.66 (1.74)			
> 50th to ≤ 75th percentile	44	6.51 (1.45)			
> 75th percentile (best sleep)	42	6.18 (2.09)			
Phase 3 cohort					
5-D Itch total score	848	–	–	128.80	< 0.0001
≤ 25th percentile (best)	220	6.25 (1.12)			
> 25th to ≤ 50th percentile	273	6.80 (1.23)			
> 50th to ≤ 75th percentile	154	7.55 (1.20)			
> 75th percentile (worst)	201	8.40 (1.21)			
Skindex-10 total score	848	–	–	63.51	< 0.0001
≤ 25th percentile (least itch)	218	6.30 (1.20)			
> 25th to ≤ 50th percentile	225	7.10 (1.41)			
> 50th to ≤ 75th percentile	206	7.37 (1.23)			
> 75th percentile (worst itch)	199	8.02 (1.34)			

Differences in weekly mean WI-NRS scores by known groups were evaluated by linear model ANOVA or t-test

MOS, Medical Outcomes Study; PGI-S, Patient Global Impression of Worst Itch Severity; SD, standard deviation; WI-NRS, Worst Itching Intensity Numerical Rating Scale

### Threshold of meaningful change

For the pooled phase 3 cohorts, the mean change in WI-NRS associated with a change from baseline to ‘minimally improved’ on the PGI-C was − 1.85 points (26% change; Table 5). Based on the secondary anchor-based approach (representing larger changes), the mean change in WI-NRS associated with a change to a much improved response on the PGI-C was − 3.54 points (51% change). The mean WI-NRS change associated with a change to minimally or much improved on the PGI-C was − 2.72 points (39% change). Mean WI-NRS change values for each PGI-C category are given in Additional file 1: Table S2.

**Table 5:** Meaningful change thresholds for WI-NRS (phase 3 cohort)

Criteria	N	Mean WI-NRS change score^a^ (SD)	Mean % change from baseline	Effect size (Cohen’s d)
Primary anchor-based approach				
PGI-C minimally improved	198	− 1.85 (1.73)	− 25.73	1.09
Secondary anchor-based approach				
PGI-C much improved	209	− 3.54 (2.08)	− 51.02	2.04
PGI-C minimally or much improved	407	− 2.72 (2.09)	− 38.72	1.48

PGI-C, Patient global impression of change; SD, standard deviation; WI-NRS, Worst Itching Intensity Numerical Rating Scale

^a^Change from baseline to end of treatment

### Exit interviews

#### Participant characteristics

Exit interviews were conducted with 70 patients in the US completing the phase 3 trials. Stratification targets of 10–20 patients by range of point reduction on the WI-NRS were met for all subgroups, except for the ≥ 3 to < 4-point reduction subgroup (n = 9). Forty-seven interviews were conducted in English and 23 in Spanish. Participants were mostly White (n = 42, 60.0%) and male (n = 46, 65.7%), and had a mean age of 55.7 ± 12.1 years (Table 2). Eight (11%) completed the interview after the specified interview window of 1–3 days after the first visit of Week 13 in the trial. One participant only answered questions related to her general itch experience, ended the study before the quantitative questionnaires were completed or debriefed, and could not be reached in follow-up attempts.

Baseline WI-NRS scores recorded in the trial ranged from 4 to 10 (Additional file 1: Table S3). Most participants had experienced baseline to Week 12 WI-NRS improvement scores ≥ 4 points (n = 26, 37.1%), followed by those who had improvement scores of ≥ 2 to < 3 (n = 18, 25.7%), ≥ 1 to < 2 (n = 10, 14.3%), ≥ 3 to < 4 (n = 9, 12.9%), ≥ 0 to < 1 (n = 5, 7.1%), and < 0 (n = 2, 2.9%).

#### Evaluation and discussion of meaningful change

**Fig. 1:**
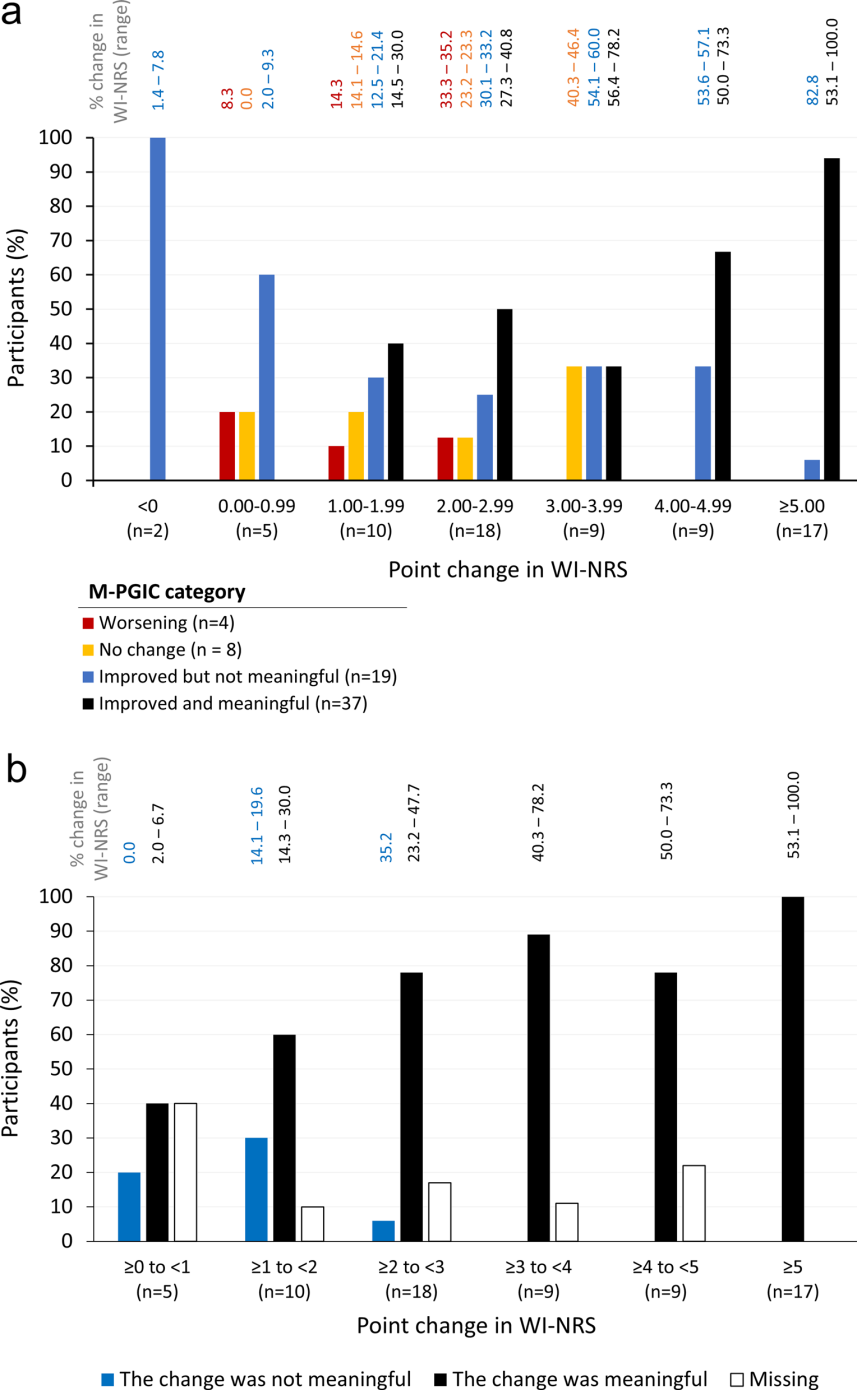
Evaluation of meaningful within-patient change on the WI-NRS in exit interviews. **a** Exit interview M-PGIC responses by WI-NRS change score. **b** WI-NRS scores by participant response on whether change was clinically meaningful. Participants who reported worsening itch over the trial were not asked if change was or was not meaningful. Abbreviations: M-PGIC, modified Patient Global Impression of Change; WI-NRS, Worst Itching Intensity Numerical Rating Scale

For the M-PGIC completed during the interview, most participants reported reduced itch and that the amount of improvement was meaningful to them (n = 37/70, 52.9%). All participants with WI-NRS changes < 1 point reported on the M-PGIC that the change experienced in itch was either not meaningful to them, or that there was no change or worsening (n = 7; Fig. 1a). Half of respondents with a WI-NRS change of ≥ 2 to < 3 points (8/16, 50.0%) and most with a change ≥ 3 points (25/35, 71%) indicated the improvement was meaningful on the M-PGIC.

When given the opportunity to review their WI-NRS change score over the course of the trial, most participants who responded indicated that their change on the WI-NRS was meaningful (n = 54/59, 92%; Fig. 1b). This included 67% of respondents (n = 6/9) with ≥ 1 to < 2-point WI-NRS changes, 93% (n = 14/15) with ≥ 2 to < 3-point changes, and all respondents (n = 32/32) with WI-NRS changes ≥ 3 points. While reviewing the WI-NRS results, 18 participants who had not reported meaningful change on the M-PGIC changed their responses and said that the change on the WI-NRS was meaningful. Thus, the distribution of participants reporting meaningful improvement differed between the M-PGIC responses and WI-NRS point-change consideration.

Participants described similar reasons for selecting the M-PGIC category of meaningful improvement – most typically reductions in frequency (e.g., “*in the first week, I started to notice that the itching was less frequent*”), intensity (e.g., “*I mean I still itch every day, but it’s not as bad*”), and duration of itch, leading to HRQoL improvements such as improved mood, increased focus, and improved sleep (e.g., “*I can lay in the bed and I can go to sleep and the itching now does not wake me up in my sleep*”). Those who experienced improvement but considered it not meaningful described reduced frequency, severity, or duration of itch but described that the improvements were intermittent, for example, only on dialysis days.

Participants who reported their WI-NRS change score was meaningful indicated noticing their itch improving (n = 39/55, 71%). For example, participants noted reduced itch frequency (n = 25/55, 45%), general itch reduction (n = 12/55, 22%), and decreased severity (n = 7/55, 13%). Some participants also described not feeling as embarrassed or self-conscious in public (n = 7/55, 13%), physical improvements on their skin as it healed (n = 6/55, 11%), and improved quality of life or state of mind (n = 6/55, 11%). Of the five participants who reported their WI-NRS change score was not meaningful, two specified that they were still experiencing itch, two said the change was not great enough for them to consider it meaningful, and one described no change in itch at all.

## Discussion

While several PROs have been developed to assess itch, few have been validated for use in clinical trials of patients with CKD-aP [8, 34], and none have had the threshold of meaningful improvement determined in these patients. Here, using a mixed methods approach, we showed the WI-NRS to be a reliable and valid PRO measure for CKD-aP. Moreover, the findings were confirmed across several large patient cohorts that together represent an international population. The content validity interviews indicated patients found the WI-NRS relevant, and that the item wording, response options, and recall period were appropriate for capturing the experiences of patients with CKD-aP. Test–retest reliability over two weeks for the WI-NRS was strong (ICCs > 0.75) [30] in both clinical trial cohorts, and is comparable to that for other PROs used to assess itch intensity in patients with chronic itch [35, 36]. Although no anchor was available to define stable itch in the phase 3 cohort test–retest analyses, ICCs > 0.80 at the discrete test–retest time points indicated enough stability in the sample (which included placebo patients) and good test–retest reliability. The construct validity analysis indicated the measure correlated well with the Skindex-10 and 5-D Itch measures, especially with conceptually related domains within those measures. The anchor-based analyses of the phase 3 cohort support that an improvement from baseline of ≥ 3 points represents an appropriate definition of meaningful within-patient change on the WI-NRS. This validates our previous findings for the phase 2 cohort, where equally a ≥ 3-point meaningful within-patient change threshold in WI-NRS was identified in quantitative distribution- and anchor-based methods [13].

A key strength to our study was the inclusion of exit interviews to confirm patients’ perspectives of what constituted a meaningful within-patient change on the WI-NRS [22]. These exit interviews used novel qualitative methodology, leveraging the weekly mean WI-NRS data from baseline and Week 12 of the clinical trials and exploring change categories by M-PGIC. Further, we used a second methodology, where we shared with participants their actual WI-NRS score changes and asked them to discuss whether or not this point change represented a meaningful change. This allowed participants to reflect and comment on their actual lived experience, as opposed to being asked to provide feedback on a hypothetical scenario [20]. In the exit interviews, when reviewing actual WI-NRS change scores experienced, all patients with a change ≥ 3 points considered the change meaningful, mentioning reduced intensity, frequency, and duration of itch and improvements in HRQoL. However, meaningful changes were also reported by two-thirds of participants with score changes in the range 1–1.99-points, suggesting changes on the WI-NRS do not have to be large in this population. This indicates both that there are individual differences in the magnitude of change considered meaningful by patients and that many patients with CKD-aP will experience meaningful improvements with changes below the ≥ 3-point change threshold.

In the exit interviews, the distribution of participants reporting meaningful improvement in their itch intensity differed between the M-PGIC responses and WI-NRS point-change consideration. This could be due to differences in the tasks asked of patients: patients could have interpreted the M-PGIC method and question to refer to their global experience related to itch in the clinical trial, whereas reviewing the WI-NRS change score may have been viewed as more specific to improvements in itch intensity. Also, some differences might be expected in patients’ responses between a 4-option categorical scale and an 11-point NRS. The order of administration of the two methods may also have influenced the results.

Although enrollment was stratified by WI-NRS point change to best represent the wider trial population completing the 12-week treatment period, patients in the exit interviews may not fully represent the real-world population since the trials included only patients with moderate-to-severe CDK-aP, whereas many patients have milder itch [1, 7–9].

## Conclusions

In conclusion, the results from this study add to evidence supporting the reliability, validity, and responsiveness of the WI-NRS for measuring itch intensity in patients with CKD-aP undergoing hemodialysis. The WI-NRS may therefore be used to assess the efficacy of anti-pruritic treatments, and potentially in clinical evaluation and management of pruritus in this population. These results are strengthened through two separate analyses: one conducted in a phase 2 trial cohort and a confirmatory analysis in a larger pooled cohort of phase 3 trial patients. The proposed, conservative ≥ 3-point reduction on the WI-NRS represents a meaningful within-patient change threshold that can be used to interpret results from clinical trials involving patients undergoing hemodialysis with moderate-to severe pruritus, for example to identify responders and non-responders to treatment.

## Supplementary Information

**Additional file 1**. **Fig. S1**. The Worst Itching Intensity Numerical Rating Scale. **Table S1**. Test-retest reliability of the WI-NRS. **Table S2**. Meaningful change thresholds for WI-NRS by PGI-C category (phase 3 cohort). **Table S3**. Baseline WI-NRS, change in WI-NRS, and M-PGIC in exit interview cohort (N=70)

## Data Availability

The datasets used and analysed during this study are available from the corresponding author on reasonable request. The data are not publicly available due to privacy or ethical restrictions.

## Abbreviations

CKD-aP: Chronic kidney disease-associated pruritus
COA: Clinical outcome assessment
HRQoL: Health-related quality of life
ICC: Intraclass correlation coefficient
M-PGIC: Modified Patient Global Impression of Change
NRS: Numerical Rating Scale
PGI-C: Patient Global Impression of Change
PGI-S: Patient Global Impression of Worst Itch Severity
PRO: Patient-reported outcome
WI-NRS: Worst Itching Intensity Numerical Rating Scale
