# Supplementary material for: Psychometric validation and meaningful change thresholds of the Worst Itching Intensity Numerical Rating Scale for assessing itch in patients with chronic kidney disease-associated pruritus
Source: J Patient Rep Outcomes. 2021 Dec 24;5:134. doi: 10.1186/s41687-021-00404-z (PMC8709801; doi:10.1186/s41687-021-00404-z)
Supplement: Supplementary file 1 — Additional file 1. Fig. S1. The Worst Itching Intensity Numerical Rating Scale. Table S1. Test-retest reliability of the WI-NRS. Table S2. Meaningful change thresholds for WI-NRS by PGI-C category (phase 3 cohort). Table S3. Baseline WI-NRS, change in WI-NRS, and M-PGIC in exit interview cohort (N=70) [file 41687_2021_404_MOESM1_ESM.docx]

# Supplementary Information


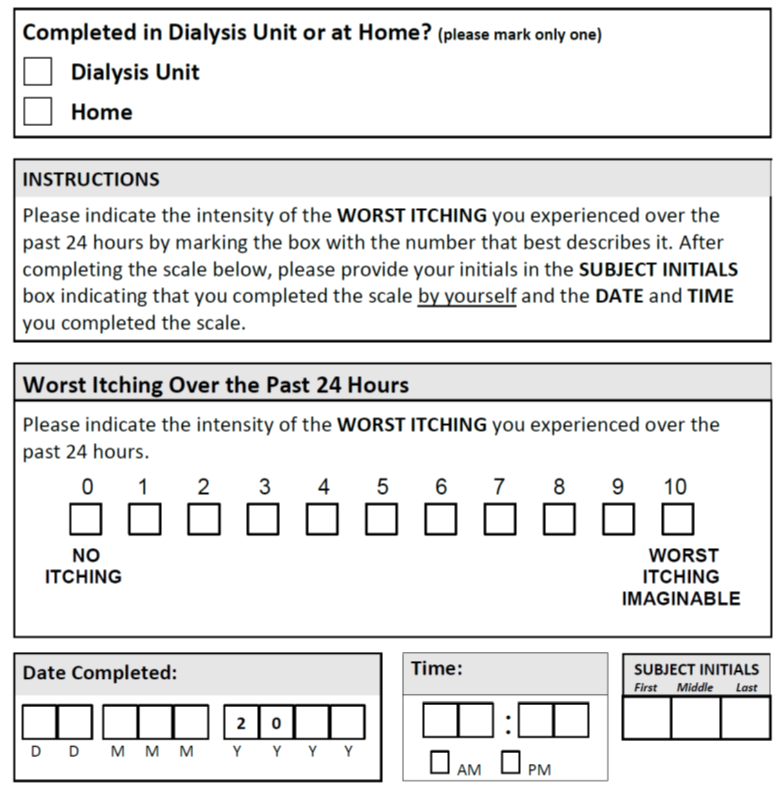


**Figure S1.** **The Worst Itching Intensity Numerical Rating Scale.**

**Table S1.** **Test-retest reliability of the WI-NRS**

| **Test-retest time points** | **Mean (SD)** | **Difference in means (SD)** | **T-value** | **P-value** | **ICC** |
| --- | --- | --- | --- | --- | --- |
| **Phase 2 cohort** |  |  |  |  |  |
| Week 1–Week 2 (N=66) |  |  |  |  |  |
| Week 1 | 6.39 (1.61) | – | – | – | – |
| Week 2 | 5.80 (1.83) | –0.60 (1.09) | –4.43 | <0.0001 | 0.756 |
| Week 2–Week 4 (N=71) |  |  |  |  |  |
| Week 2 | 5.19 (1.95) | – | – | – | – |
| Week 4 | 4.44 (2.21) | –0.76 (1.12) | –5.73 | <0.0001 | 0.805 |
|  |  |  |  |  |  |
| **Phase 3 cohort (pooled)** |  |  |  |  |  |
| Week 1–Week 2 (N=811) |  |  |  |  |  |
| Week 1 | 6.48 (1.83) | – | – | – | – |
| Week 2 | 5.92 (2.12) | –0.56 (1.14) | –13.9 | <0.0001 | 0.802 |
| Week 2–Week 4 (N=780) |  |  |  |  |  |
| Week 2 | 5.92 (2.13) | – | – | – | – |
| Week 4 | 5.31 (2.39) | –0.61 (1.29) | –13.3 | <0.0001 | 0.808 |

Abbreviations: ICC, intraclass correlation coefficient; SD, standard deviation; WI‑NRS, Worst Itching Intensity Numerical Rating Scale

**Table S2.** **Meaningful change thresholds for WI-NRS by PGI-C category
(phase 3 cohort)**

| **PGI-C category** | **N** | **Mean WI-NRS change score^a^ (SD)** | **Mean % change from baseline** |
| --- | --- | --- | --- |
| Very much improved | 140 | –5.09 (2.11) | –70.69 |
| Much improved | 209 | –3.54 (2.08) | –51.02 |
| Minimally improved | 198 | –1.85 (1.73) | –25.73 |
| No change | 128 | –0.61 (1.41) | –7.72 |
| Minimally worse | 21 | –0.45 (1.87) | –2.87 |
| Much worse | 9 | –0.12 (1.96) | 1.53 |
| Very much worse | 5 | 0.20 (1.69) | 3.16 |
|  |  |  |  |
| Missing | 10 | – | – |

Abbreviations: PGI-C, Patient Global Impression of Change; SD, standard deviation; WI‑NRS, Worst Itching Intensity Numerical Rating Scale

^a^ Change from baseline to end of treatment

**Table S3.** **Baseline WI-NRS, change in WI-NRS, and M-PGIC in exit interview cohort (N=70)**

| **Item** | **n (%)** |
| --- | --- |
| Baseline NRS |  |
| ≥4 to <6 | 7 (10.0) |
| ≥6 to <8 | 34 (48.6) |
| ≥8 to 10 | 29 (41.4) |
| Baseline to Week 12 change in NRS |  |
| <0 | 2 (2.9) |
| ≥0 to <1 | 5 (7.1) |
| ≥1 to <2 | 10 (14.3) |
| ≥2 to <3 | 18 (25.7) |
| ≥3 to <4 | 9 (12.9) |
| ≥4 to <5 | 9 (12.9) |
| ≥5 | 17 (24.3) |
| M-PGIC |  |
| My itch got worse | 4 (5.7) |
| No change | 8 (11.4) |
| My itch got better but the amount of improvement was not meaningful to me | 19 (27.1) |
| My itch got better and the amount of improvement was meaningful to me | 37 (52.9) |
| Missing^a^ | 2 (2.9) |

Abbreviations: M-PGIC, modified Patient Global Impression of Change; WI-NRS, Worst Itching Intensity Numerical Rating Scale

^a^ Two participants marked multiple responses; both participants’ responses were set to missing
